# Can wearable technology be used to approximate cardiopulmonary exercise testing metrics?

**DOI:** 10.1186/s13741-021-00180-w

**Published:** 2021-03-16

**Authors:** Laura Jones, Laura Tan, Suzanne Carey-Jones, Nathan Riddell, Richard Davies, Ashleigh Brownsdon, Mark Kelson, Rhys Williams-Thomas, Monica Busse, Michael M. Davies, Matt P. G. Morgan

**Affiliations:** 1 Adult Critical Care Unit, University Hospital of Wales, Cardiff, CF14 4RQ UK; 2 Department of Anaesthetics, University Hospital of Wales, Cardiff, UK; 3 Department of Mathematics, University of Exeter, Exeter, UK; 4 Department of Mathematics, University of Exeter, Exeter, UK; 5 Centre for Trials Research, Cardiff University, Cardiff, UK; 6 Department of Colorectal Surgery, University Hospital of Wales, Cardiff, UK

**Keywords:** Cardiopulmonary exercise testing, VO_2_, Anaerobic threshold, Perioperative medicine, Wearable technology

## Abstract

**Background:**

Consumer wrist-worn wearable activity monitors are widely available, low cost and are able to provide a direct measurement of several markers of physical activity. Despite this, there is limited data on their use in perioperative risk prediction. We explored whether these wearables could accurately approximate metrics (anaerobic threshold, peak oxygen uptake and peak work) derived using formalised cardiopulmonary exercise testing (CPET) in patients undergoing high-risk surgery.

**Methods:**

Patients scheduled for major elective intra-abdominal surgery and undergoing CPET were included. Physical activity levels were estimated through direct measures (step count, floors climbed and total distance travelled) obtained through continuous wear of a wrist worn activity monitor (Garmin Vivosmart HR+) for 7 days prior to surgery and self-report through completion of the short International Physical Activity Questionnaire (IPAQ). Correlations and receiver operating characteristic (ROC) curve analysis explored the relationships between parameters provided by CPET and physical activity.

**Device selection:**

Our choice of consumer wearable device was made to maximise feasibility outcomes for this study. The Garmin Vivosmart HR+ had the longest battery life and best waterproof characteristics of the available low-cost devices.

**Results:**

Of 55 patients invited to participate, 49 (mean age 65.3 ± 13.6 years; 32 males) were enrolled; 37 provided complete wearable data for analyses and 36 patients provided full IPAQ data.

Floors climbed, total steps and total travelled as measured by the wearable device all showed moderate correlation with CPET parameters of peak oxygen uptake (peak VO_2_) (*R* = 0.57 (CI 0.29–0.76), *R* = 0.59 (CI 0.31–0.77) and *R* = 0.62 (CI 0.35–0.79) respectively), anaerobic threshold (*R* = 0.37 (CI 0.01–0.64), *R* = 0.39 (CI 0.04–0.66) and *R* = 0.42 (CI 0.07–0.68) respectively) and peak work (*R* = 0.56 (CI 0.27–0.75), *R* = 0.48 (CI 0.17–0.70) and *R* = 0.50 (CI 0.2–0.72) respectively).

Receiver operator curve (ROC) analysis for direct and self-reported measures of 7-day physical activity could accurately approximate the ventilatory equivalent for carbon dioxide (V_E_/VCO_2_) and the anaerobic threshold. The area under these curves was 0.89 for V_E_/VCO_2_ and 0.91 for the anaerobic threshold. For peak VO_2_ and peak work, models fitted using just the wearable data were 0.93 for peak VO_2_ and 1.00 for peak work.

**Conclusions:**

Data recorded by the wearable device was able to consistently approximate CPET results, both with and without the addition of patient reported activity measures via IPAQ scores. This highlights the potential utility of wearable devices in formal assessment of physical functioning and suggests they could play a larger role in pre-operative risk assessment.

**Ethics:**

This study entitled “uSing wearable TEchnology to Predict perioperative high-riSk patient outcomes (STEPS)” gained favourable ethical opinion on 24 January 2017 from the Welsh Research Ethics Committee 3 reference number 17/WA/0006. It was registered on ClinicalTrials.gov with identifier NCT03328039.

## Background

Cardiopulmonary exercise testing (CPET) is a non-invasive clinical tool that allows evaluation of exercise capacity by global assessment of cardiorespiratory function. Using measures of respiratory oxygen uptake (VO_2_), carbon dioxide production (VCO_2_) and ventilatory measures, it enables objective evaluation of both submaximal and peak exercise responses (Wu et al. 2017). CPET is routinely used to measure functional capacity in patients prior to major surgery in order to aid clinical decision-making regarding a patient’s suitability for surgery and inform risk assessment. Information obtained from CPET can be used to estimate the likelihood of perioperative morbidity and mortality, to direct preoperative interventions and optimisation, guide decisions regarding perioperative management and inform choice of post-operative care (ward vs. critical care) (Levett et al. 2018). There is increasing interest in the use of consumer wearable devices to approximate physical activity parameters for use in the healthcare setting.

However, CPET is expensive and requires a trained operator, specialist equipment and a significant time investment from both medical staff and the patient, which limit its availability for routine use. In addition, there are several absolute contraindications to CPET, including many cardiac and respiratory conditions, that may result in some patients being ineligible for testing (Albouaini et al. 2007). Some patients may not be able to perform CPET or achieve a maximal CPET due to frailty or arthritic joints. Nosocomial infection, especially currently with COVID-19, is another reason where encouraging community access to testing may be preferable. While the inability to perform CPET does indeed provide information, it provides it in a binary format, which conveys less statistical information that the more graded response achievable from a wearable, which meets individuals where they are.

The use of consumer wrist-worn wearable activity monitors is increasing due to their widespread availability, decreasing cost and improving accuracy (Rochmis and Blackburn 1971). These continuously-evolving technologies are able to provide direct measurement of several markers of physical activity and their growing popularity highlights the need to further evaluate their utility in the healthcare setting. These devices are able to directly record large amounts of activity data without a physician present and represent a possible alternative to CPET that is more accessible, more affordable and may better represent a patient’s day-to-day functional capacity.

The aim of this study (uSing wearable TEchnology to Predict perioperative high-riSk patient outcomes (STEPS)) was to assess the feasibility of collecting activity and physiological data of sufficient quantity and quality using wrist-worn activity monitors. We then further explored the relationship between the data collected via these wearable activity measures and pre-operative CPET.

## Methods

### Study population

Patients scheduled for major elective intra-abdominal surgery at the University Hospital of Wales between June 2017 and February 2018 were included in the study. Inclusion criteria included: aged 18 years or older, capacity to consent and a clinical indication for planned CPET before elective major surgery. Exclusion criteria included atrial fibrillation, nickel allergy, unable to wear a watch, unable to undergo CPET and pregnancy. Baseline information on age, gender and body mass index (BMI) were recorded. Information about the study was sent by post along with the CPET appointment. Patients had a discussion about the study on the day of attending their CPET appointment and consent taken prior to their test.

Our original sample size was chosen pragmatically to be 100. This would have allowed the estimation of any feasibility proportion to within at least plus or minus 9.8 percentage points using a 95% confidence interval.

### CPET

Preoperative CPET was conducted and interpreted by a consultant anaesthetist experienced in CPET in accordance with national guidelines (Levett et al. 2018) using an electromagnetically braked cycle ergometer (Lode, Gronigen, The Netherlands) and a Medgraphics Ultima metabolic cart (MedGraphics, Gloucester, UK). Calibration was undertaken in accordance with manufacturer's guidelines using a 3-L syringe (Hans Rudolph, Kansas City, KS, USA) and reference calibration gases. During data collection, the middle five of seven breaths were averaged. An exercise protocol was used whereby patients cycled at 60 rpm. for 3 min in an unloaded freewheeling state, followed by a progressively ramped period of exercise (from 5 to 15 W min−1 based on mass, stature, age, and sex) to volitional or symptom-limited termination, followed by 3 min recovery. Medgraphics Breeze software automatically determined peak oxygen uptake (*V*O2peak; defined as the highest O2 uptake during the final 30 s of exercise reported). The AT was manually interpreted using the V-slope method (Beaver, Wassermen, and Whipp, 1986) and supported by comparison of end-tidal oxygen tension (ETO2) and ventilatory equivalent for oxygen (*VE/VO*_*2*_) plots. The ventilatory equivalent for carbon dioxide (*VE/V*CO2 ) was identified at the AT or was recorded as the gradient of the linear *VE/V*CO2 relationship if the AT could not be identified.

AT was not recorded if AT was not reached during the test or the respiratory exchange ratio (RER) was persistently > 1.0 during the exercise test precluding the determination of AT.

### Study outcomes

All participants underwent CPET as part of their routine pre-operative workup. Four key CPET measures of activity were recorded: peak oxygen consumption (peak VO_2_), the ventilatory equivalent for carbon dioxide (V_E_/VCO_2_ slope), the anaerobic threshold (AT) and peak work (also known as peak power output). The following thresholds were used: 14 ml/kg/min was used for the peak VO_2_ threshold*,* 34 ml/min for the V_E_/VCO_2_ slope and 11 ml/kg/min for the anaerobic threshold (Mück et al. 2019). A median split was used to divide recorded peak work values.

In addition to this, all participants wore a wearable device continuously for 7 days prior to their surgery. The wearable device used was the Garmin Vivosmart HR+ smart activity tracker. A participant information leaflet was sent to all eligible patients one week prior to attending a routine CPET clinic. The devices were then issued to participants after they had completed their CPET assessment. The devices were worn continuously for the 7-day study duration and were then removed and stored until return during the next routine clinic appointment. Patients were given clear advice regarding wearing the device and issued with a charger in case of power failure, although the battery of the device was sufficient for 7 days of continuous use. The device recorded resting heart rate, average heart rate, maximum heart rate, total steps, floors climbed, number of intense minutes of exercise, total calories and total distance travelled. These variables were averaged across the 7-day period prior to analysis.

After the 7 days, participants completed the International Physical Activity Questionnaire (IPAQ) which identified the metabolic equivalent task (MET) minutes achieved in different domains (work, transportation, domestic, garden, leisure time) by self-report.

The total MET minutes of physical activity per week was computed by summing the MET minutes from each category (walking, moderate or vigorous activity).

The total time per week in each category was then multiplied by a constant depending on the level of intensity of the exercise: 3.3 for walking, 4.0 for moderate-intensity activity and 8.0 for vigorous-intensity activity. Once the MET minutes per week for each category were computed, these were summed to produce the total physical activity MET-minutes/week and the IPAQ global score; the patients were then categorised as having a high, moderate or low level of physical activity.

### Statistical analysis

Descriptive data is presented as mean ± standard deviation. Statistical modelling was used to explore the relationship between parameters provided by CPET, self-reported activities via the IPAQ and activity measurements recorded by the wearable device.

Graphical exploration of the wearable data was performed, alongside range checks.

Incomplete data capture was possibly due to sub-optimal fit of the device or movement of the device on the wrist during capture, with the device documentation suggesting that a issues such as sweat, lotion or sunscreen on the wrist, fit of device (location on the wrist and tightness of the strap) and intensity of the activity can contribute to limitations of data capture. In the future, these issues could be address by repeated checks to ensure correct fit and care of the device at all times and continued patient education on optimal use of the device.

Correlations between variables were calculated using Pearson correlation coefficients. With Pearson correlation assumptions met, via scatter plots demonstrating linear covariation and visual inspection of Q-Q plots. Linear regression was used to explore whether the eight wearable variables could be used to estimate values for the four CPET measures of activity. In addition, further analysis explored whether the addition of IPAQ global scores could improve this estimation. Three models were fitted for each of the four measures of CPET activity, with all including basic demographic variables (age, gender, BMI). The first model used only the eight wearable values as predictors, the second used only the global IPAQ score and the third model used both of these components together.

The models were compared using the Akaike Information Criteria (AIC). Standard model diagnostics were explored to ensure adequate model fit, including fitted versus residual plots. Models were assessed and compared using appropriate statistics, including *R*^2^ and adjusted *R*^2^ values, the percentage of correct predictions and Pearson correlation coefficients (with associated 95% confidence intervals) between the fitted and observed values. Receiver operator characteristic (ROC) curves for these models were also compared.

## Results

Of 55 invited to participate, 49 (mean age 65 ± 13.6 years, 17 females) were included in the study. All participants (*n* = 49) underwent CPET prior to surgery but only 40 participants had values recorded for their anaerobic threshold. Only 36 participants completed the IPAQ. All wearable devices were returned; there was no damage noted in any of the devices and no reported issues with the devices during the study but of the 49 devices returned, 12 demonstrated incomplete data capture and were excluded from analysis. This was not due to device removal but rather failure of the device to record data at some timepoints. Correlations between CPET, wearable and IPAQ variables were only calculated for individuals who had complete data for each variable required in the analysis.

Mean (SD) VO_2_ max was 18.2 ± 4.5 ml/kg/min, V_E_/VCO_2_ 31.7 ± 4.5, peak work 105.9 ± 39.2 W and anaerobic threshold 11.7 ± 2.4 ml/kg/min. Mean 7-day step count was 6040 ± 3323 steps/day and average heart rate was 64 ± 6.8 bpm.

With regard to mean ± SD self-reported MET minutes, 41.7% proportion of the study sample categorised as high physical activity (MET total 6898 ± 2847), 30.6% moderate (1739 ± 516) and 27.8% low physical activity (519 ± 565).

**Fig. 1:**
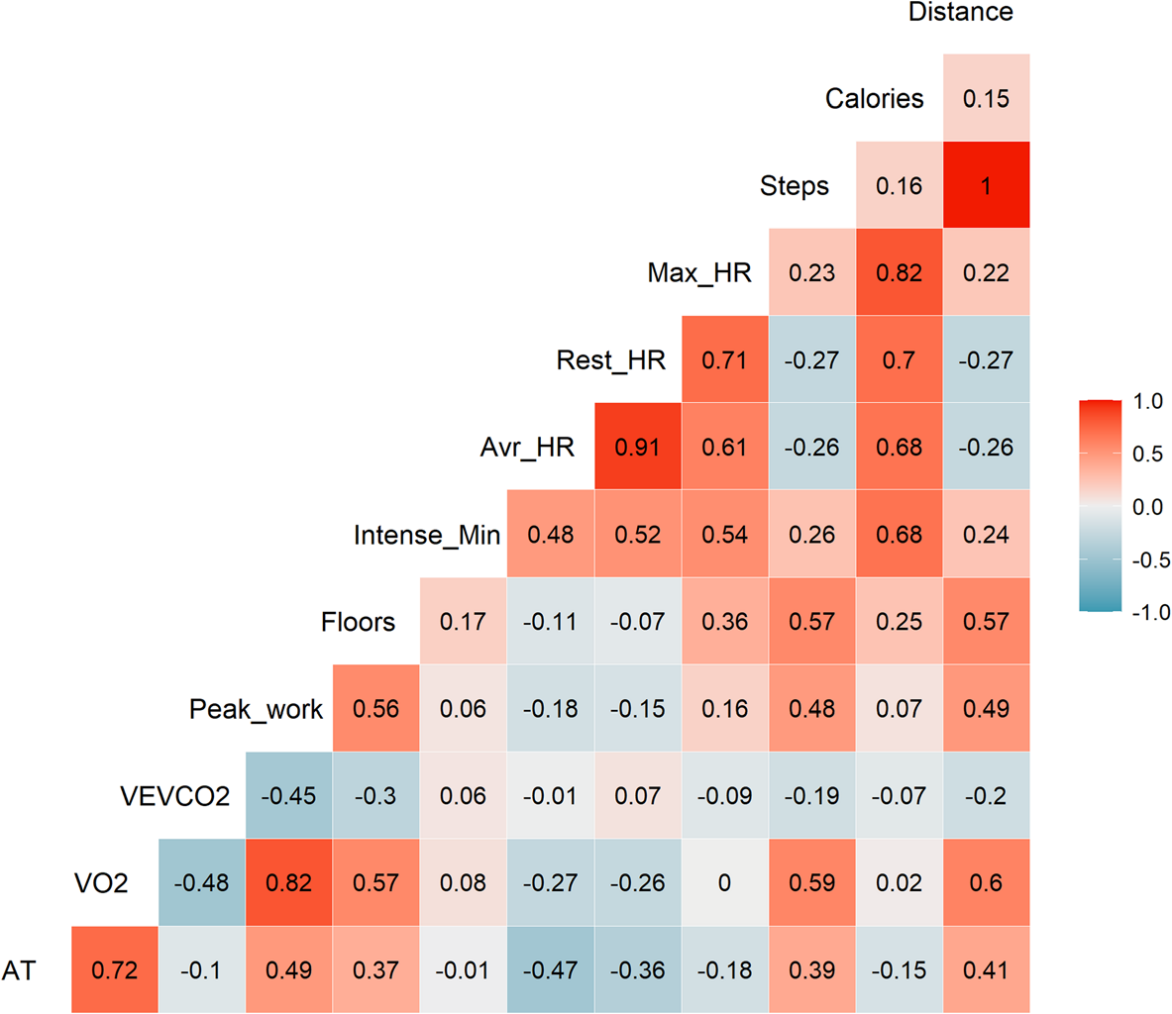
Pearson correlation coefficients between the CPET parameters and the wearable parameters. CPET parameters are peak work, VE/VCO2, VO2, AT (anaerobic threshold). Wearable parameters are floors climbed, intense minutes of activity, average heart rate, resting heart rate, maximum heart rate, step count, total calories and total distance

Floors climbed, total number of steps and total distance travelled (derived from number of steps) as recorded by the wearable device were most correlated with the CPET parameters (Table 1, Fig. 1). Floors climbed, total steps and total distance travelled all showed moderate correlation with peak VO_2_ (*R* = 0.57, *R* = 0.59 and *R* = 0.62 respectively), anaerobic threshold (*R* = 0.37, *R* = 0.39 and *R* = 0.42 respectively) and peak work (*R* = 0.56, *R* = 0.48 and *R* = 0.50 respectively). These three wearable parameters were also significantly correlated with IPAQ global scores.

**Table 1:** Pearson correlation coefficients, and 95% confidence intervals, between the measured values for the four CPET scores and the IPAQ global score, and the eight wearable variables

Wearable variable	CPET variables				IPAQ global score
	Peak VO2	VE/VCO2	AT	Peak work	
**Floors climbed**	0.57 (0.29, 0.76)	− 0.30 (− 0.58, 0.04)	0.37 (0.01, 0.64)	0.56 (0.27, 0.75)	0.71 (0.45, 0.86)
**Intense minutes**	0.08 (− 0.26, 0.41)	0.06 (− 0.29, 0.39)	− 0.01 (− 0.37, 0.35)	0.06 (− 0.28, 0.39)	0.32 (− 0.07, 0.63)
**Average heart rate**	− 0.27 (− 0.55, 0.08)	− 0.01 (− 0.35, 0.33)	− 0.47 (− 0.71, − 0.13)	− 0.18 (− 0.49, 0.17)	− 0.16 (− 0.51, 0.24)
**Resting heart rate**	− 0.26 (− 0.55, 0.08)	0.07 (− 0.28, 0.40)	− 0.36 (− 0.64, 0.00)	− 0.15 (− 0.46, 0.20)	− 0.09 (− 0.46, 0.30)
**Maximum heart rate**	0.00 (− 0.34, 0.34)	− 0.09 (− 0.42, 0.25)	− 0.18 (− 0.50, 0.20)	0.16 (− 0.19, 0.47)	0.31 (− 0.08, 0.62)
**Total steps**	0.59 (0.31, 0.77)	− 0.19 (− 0.50, 0.16)	0.39 (0.04, 0.66)	0.48 (0.17, 0.70)	0.54 (0.20, 0.76)
**Total calories**	0.02 (− 0.32, 0.36)	− 0.07 (− 0.40, 0.27)	− 0.15 (− 0.48, 0.22)	0.07 (− 0.27, 0.40)	0.20 (− 0.20, 0.54)
**Total distance**	0.62 (0.35, 0.79)	− 0.21 (− 0.51, 0.14)	0.42 (0.07, 0.68)	0.50 (0.20, 0.72)	0.51 (0.17, 0.75)

*AT* Anaerobic threshold, *CPET* Cardio-pulmonary exercise testing, *VO2* Maximal oxygen uptake, *IPAQ* International Patient Activity Questionnaire

A slower average heart rate and resting heart rate were associated with improved anaerobic threshold (*R* = − 0.47 and *R* = − 0.36) and peak VO_2_ (*R* = − 0.27 and *R* = − 0.26). None of the wearable variables were strongly correlated with V_E_/VO_2_. There were no strong correlations observed between intense minutes of activity, maximum recorded heart rate or total calories burned with any of the CPET parameters. Of note, the average, resting and maximum heart rate variables had small standard deviations relative to their mean values. These small standard deviations may partially explain why the correlations between the heart rate variables and the CPET measures were weaker than expected.

All correlations between the wearable measures, CPET parameters and IPAQ global scores are shown in Table 1 and depicted graphically in Fig. 1.

As shown in Table 2, using all eight of the wearable variables together in linear regression gave a stronger correlation between the measured CPET values, specifically for peak VO_2_, anaerobic threshold and peak work values. For all three models, the model fit was less accurate when estimating the V_E_/VCO_2_ slope than for the other three measures. However, the AIC values of these models cannot be directly compared as each model includes different parameters and slight variations in sample size. When comparing the *R*^2^ values, model diagnostics demonstrated that the model using both the wearable data and the IPAQ global score together was better in estimating CPET scores for all four of the CPET measures (Table 2).

**Table 2:** Model fit diagnostics for numerous linear models estimating values for the four CPET measures of activity

CPET variable	Predictor	Sample size	Number of parameters	AIC	***R***^**2**^	***R***^**2**^ adjusted	% correct	Pearson correlation (95% CI)
**Peak VO2**	Wearable	34	11	181.62	0.74	0.62	91.18	0.86 (0.74, 0.93)
	IPAQ	27	4	163.98	0.29	0.16	77.78	0.54 (0.20, 0.76)
	Both	27	12	150.89	0.76	0.55	88.89	0.87 (0.73, 0.94)
**VE/VCO2**	Wearable	34	11	219.98	0.26	− 0.11	79.41	0.51 (0.20, 0.72)
	IPAQ	27	4	172.54	0.19	0.05	85.19	0.44 (0.07, 0.70)
	Both	27	12	179.75	0.41	− 0.08	85.19	0.65 (0.35, 0.82)
**Anaerobic threshold**	Wearable	30	11	129.78	0.72	0.56	70	0.85 (0.71, 0.93)
	IPAQ	23	4	106.71	0.41	0.27	65.22	0.64 (0.30, 0.83)
	Both	23	12	89.91	0.86	0.69	78.29	0.93 (0.83, 0.97)
**Peak work**	Wearable	34	11	328.30	0.73	0.59	100	0.85 (0.72, 0.92)
	IPAQ	27	4	271.35	0.44	0.34	85.19	0.66 (0.38, 0.83)
	Both	27	12	257.24	0.82	0.66	81.48	0.90 (0.80, 0.96)

**Fig. 2:**
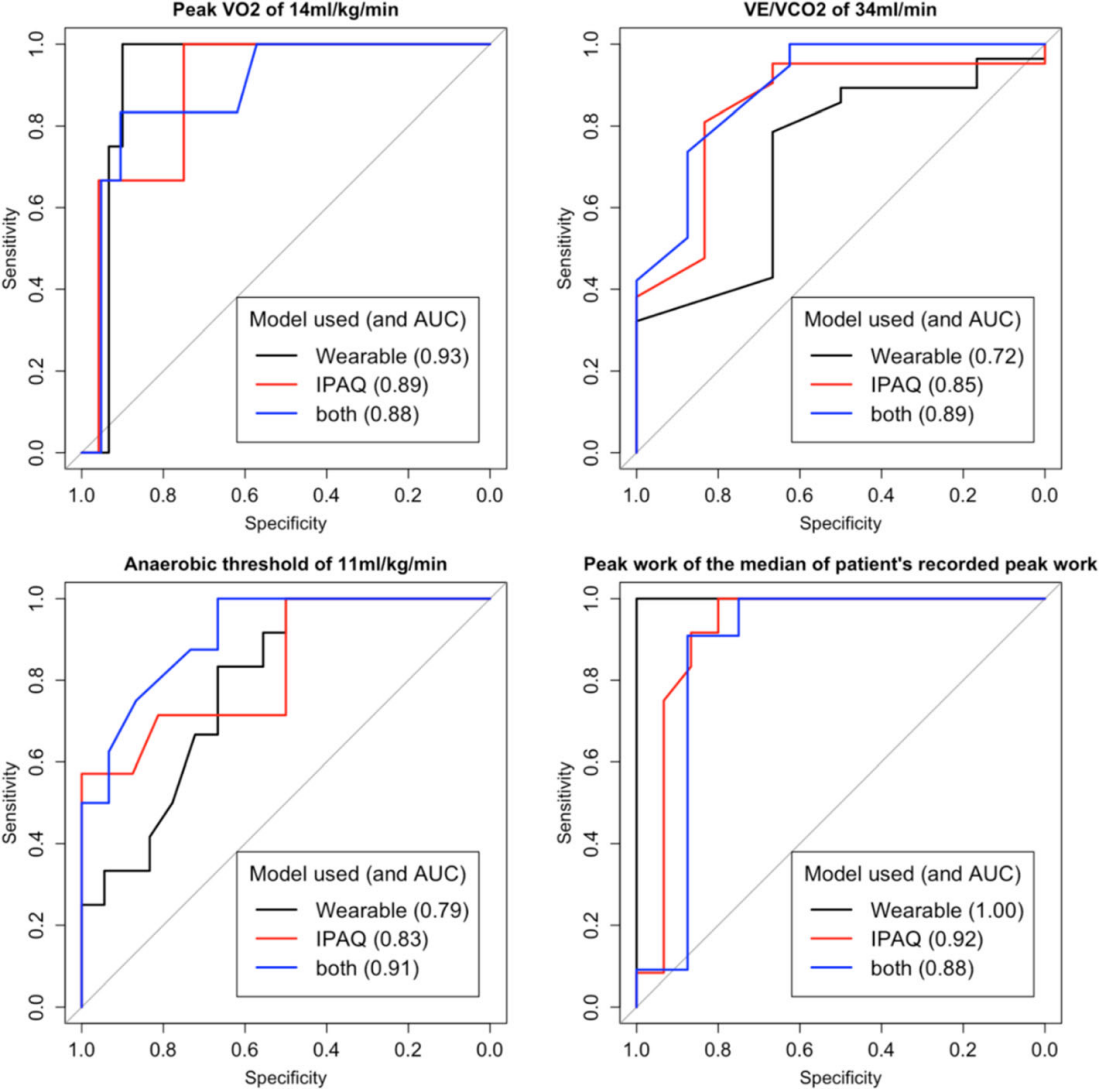
Receiver operator curve (ROC) analysis curves for the four CPET measures of activity

Receiver operator curve (ROC) analysis demonstrated that the model using both the wearable data and the IPAQ scores was the most accurate in approximating V_E_/VCO_2_ and the anaerobic threshold (Fig. 2). The area under these curves was 0.89 for V_E_/VCO_2_ and 0.91 for the anaerobic threshold. For peak VO_2_ and peak work, the ROC curves suggest that the models fitted using just the wearable data were a better classification, with the area under these curves 0.93 for peak VO_2_ and 1.00 for peak work. Nonetheless, linear regression using the wearable variables and the IPAQ global score independently, were also able to successfully estimate the CPET scores in isolation.

### Missing values

Although there was a relatively high proportion of missing data across the dataset, the correlations of wearable data with CPET values remained robust perhaps attributed to the frequency of data sampling despite missing data.

We have also analysed the results for the 9 patients missing AT and the 5 of these with missing wearable data. There were no statistical significant differences when this analysis is included.

There was a trend towards lower cardiorespiratory fitness in the no-AT group. There was lower Peak VO2, higher VE/VCO2, lower peak work from which you might infer lower cardiorespiratory fitness, but the numbers are likely too small for statistical analysis.

## Discussion

Wearable activity monitors are becoming increasingly popular amongst consumers due to their decreasing cost and increasing accessibility (Mück et al. 2019). The market for wearable devices is experiencing rapid growth year on year, with worldwide wearable devices forecasted to increase from 593 million devices globally in 2018 to 929 million devices by 2021, rising to over 1 billion devices by 2022 (Mancini et al. 1991). However, there remains a paucity of data around the role of these wearable devices in the healthcare setting, despite their great potential to improve upon efficiency, cost and patient experience.

In this study, we found that data collection using this novel method to be feasible in terms of logistical and data capture considerations. We also found a significant correlation between wearable device measurements of step count, floors climbed and total travelled as measured by a Garmin Vivosmart HR+ wearable device and CPET parameters of anaerobic threshold, peak VO_2_ and peak work. In addition, the data recorded by the wearable device was consistently associated with CPET results, both with and without the addition of patient reported activity measures via IPAQ scores. There will be times where an IPAQ will be preferred especially with individuals who struggle with the technological aspects of wearable devices.

### Limitations

No data was recorded regarding concomitant medications that patients may have been taking prior to their surgery. This is particularly relevant with regard to beta-blockers, which are commonly given to patients to manage arrhythmias and decrease heart rate. This may partly explain why only low correlations were found between the maximum heart rate as recorded by the wearable device and the CPET measures.

In line with recommended practice (cite Lancaster, Dodd and Williamson), we highlight that the results of our hypothesis testing should be regarded as exploratory and therefore interpreted with caution, as no formal power calculations have been carried out for them.

Data for each recorded parameter was extracted directly from each device. Of note, raw data captured by the device was likely cleaned and possibly adjusted by algorithms in the Garmin software prior to being made available for extraction; however, specific details of this process are not available as the software is proprietary.

No further manipulation of the data was performed by the authors following extraction with the extracted numerical values for each parameter used directly in analysis as stated in the “Methods” section.

These results also only apply to the specific wearable device tested and variation between models and further developments in the wearable technology may limit the ability to extrapolate this data accurately to all wearable devices. However, we feel the main findings are likely to remain the same as the core technology between models is similar and these findings still provide key descriptive evidence on which to base further studies.

Additional limitations of wearable devices include concerns regarding the accuracy of the parameters they record. In addition, there are large variations in quality and accuracy of recording accuracy between different makes and models of wearable device (Forecast 2019). With regard to utilisation of these devices in the healthcare setting, it would be important to identify reliable and evidence-based models of device and to ensure the devices were used correctly by patients and the data recorded was accurate. Including a debrief with the patients when the device data is available may be added to ensure correct usage and hence data. However, we feel these represent areas where caution must be taken during the integration of wearables in the medical setting, rather than insurmountable issues, and highlight the need for further studies to build a better understanding of this growing field. In addition, many devices use processed versions of the raw data and changes in these internal algorithms may impact upon future analysis. The cost of wearable devices must also be considered and weighed up against the expense of CPET, although evidence suggests that wearable technologies are continuing to decrease in cost (Shcherbina et al. 2017). Formal feasibilities studies will be needed to conclude that the continued use of wearable technology in a medical setting is appropriate.

### The future

With continuously improving technology, most smart wrist-worn devices are now not only a chronograph but are also able to monitor a wide variety of activity parameters and personal analytics, including several heart rate parameters, exercise frequency and activity intensity, as well as recording sleep parameters and heart rate variability. The market for these devices is currently increasing exponentially with their improving aesthetics, decreasing cost, further miniaturisation of components, improving battery life, and increasing number of features offered (Mück et al. 2019). As the distinction between consumer health wearables and medical devices becomes more fluid, it is important to continue to evaluate their potential benefits in the healthcare setting and explore areas in which they can be best utilised as assets to improve upon existing systems and workflows.

In light of the expense, clinical expertise required, purchase and maintenance of specialist equipment and contraindications for CPET, these findings highlight the potential utility of wearable devices as part of the preoperative assessment of functional capacity prior to major surgery. CPET can be time and labour intensive for both the patient and the medical staff who conduct the testing, as well as taking the patient into an artificial setting in order to push them to their maximal exercise limits. Wearable devices are able to integrate smoothly into a patient’s existing routine and may provide a more accurate representation of a patient’s day-to-day function within their own environment, so long as there are worn correctly. We envisage the use of wearable devices as a more widely accessible screening tool that could be used to better target CPET resources and further improve preoperative assessment.

Studies suggest that anaerobic threshold predicts postoperative complications and mortality, so the consistent correlation of anaerobic threshold with the majority of the wearable measures we analysed adds further weight to the potential of the wearable data to inform upon post-surgical outcomes (Canhoto and Arp 2017).

V_E_/VCO_2_ (the ventilatory equivalent for carbon dioxide) was shown to correlate less well with wearable parameters and IPAQ scores. This measure is the ratio of minute ventilation to CO2 output, providing information on ventilation efficiency in the lungs.

We feel a much larger study is warranted to explore if the correlations can be strengthened with a larger sample size. These studies would focus on the device measures that correlate best with CPET metrics in a high-risk surgery patient cohort where patient-centred outcome measures could be assessed.

## Conclusions

We have shown that it is possible to utilise wearable devices to approximate CPET metrics. Further studies are required to continue to evaluate the potential of wearable devices in the healthcare setting, and to further explore how these systems can be accommodated into existing practices to improve upon efficiency and patient care.

## Data Availability

The datasets supporting the conclusions of this article are available on request.

## Abbreviations

BMI: Body mass index
CPET: Cardiopulmonary exercise testing
ROC: Receiver operator curve
VO_2_: Oxygen consumption
AT: VO_2_ at anaerobic threshold
IPAQ: Physical Activity Questionnaire
AIC: Akaike Information Criteria
